# Tracking Strains in the Microbiome: Insights from Metagenomics and Models

**DOI:** 10.3389/fmicb.2016.00712

**Published:** 2016-05-19

**Authors:** Ilana L. Brito, Eric J. Alm

**Affiliations:** ^1^Department of Biological Engineering, Massachusetts Institute of TechnologyCambridge, MA, USA; ^2^Center for Microbiome, Informatics and Therapeutics, Massachusetts Institute of TechnologyCambridge, MA, USA

**Keywords:** microbiome, metagenomics, models, biological, strain diversity, genotyping techniques, bacterial genomics

## Abstract

Transmission usually refers to the movement of pathogenic organisms. Yet, commensal microbes that inhabit the human body also move between individuals and environments. Surprisingly little is known about the transmission of these endogenous microbes, despite increasing realizations of their importance for human health. The health impacts arising from the transmission of commensal bacteria range widely, from the prevention of autoimmune disorders to the spread of antibiotic resistance genes. Despite this importance, there are outstanding basic questions: what is the fraction of the microbiome that is transmissible? What are the primary mechanisms of transmission? Which organisms are the most highly transmissible? Higher resolution genomic data is required to accurately link microbial sources (such as environmental reservoirs or other individuals) with sinks (such as a single person's microbiome). New computational advances enable strain-level resolution of organisms from shotgun metagenomic data, allowing the transmission of strains to be followed over time and after discrete exposure events. Here, we highlight the latest techniques that reveal strain-level resolution from raw metagenomic reads and new studies that are tracking strains across people and environments. We also propose how models of pathogenic transmission may be applied to study the movement of commensals between microbial communities.

Since the dawn of germ theory, epidemiology has focused on pathogens, their transmission routes and the consequences of their dispersal. Only recently have we fully appreciated the diverse roles of the thousands of microbial species that inhabit the human body. It is therefore sensible to broaden our questions about transmission dynamics and transmission routes to encompass the full range of commensal organisms. Recently, it has been suggested that diseases associated with dysbioses, such as Crohn's disease, rheumatoid arthritis and multiple sclerosis, may be transmissible (reviewed in Faith et al., 2015). There is also mounting evidence that the passive transmission of commensal bacteria may carry health benefits: in preventing obesity (Mueller et al., 2015), autoimmune disease (Olszak et al., 2012), and even certain cancers (Chen and Blaser, 2007; Hung and Wong, 2009). New therapeutics involve intentionally transmitting entire gut communities to treat recurrent *Clostridium difficile* infections (Kassam et al., 2013), and may ultimately be used to treat a wider array of conditions. Despite advances in DNA sequencing that have enabled wide-scale characterizations of a large variety of microbial communities, little is known about how non-pathogenic microbes move between people and places.

For instance, we do not know what portion of the microbiome is transmissible. Research has instead focused on what *can* colonize, i.e., determining what factors impact colonization (Sonnenburg et al., 2005; Vaishnava et al., 2008; Goodman et al., 2009; Cullen et al., 2015), rather than what *does* colonize after exposure. What role does the transfer of organisms play in shaping either daily or punctuated shifts in our microbiomes? Our ability to answer these question currently relies on data from 16S marker gene surveys which can resolve differences between species. In some cases, coarse species-level data is sufficient to observe commensal transmission within the microbiome. In the gut, microbes associated with cured meat and cheese appear after ingestion (David et al., 2014a), and exogenous organisms repopulate the gut after acute gastrointestinal illness (David et al., 2014b). Likewise, contact with inanimate objects results in the transmission of commensals from our skin to proximal environments (Costello et al., 2009; Fierer et al., 2010; Lax et al., 2014). Perhaps unsurprisingly, infants are initially colonized by their mothers' skin and vaginal flora depending on birth method (Dominguez-Bello et al., 2010), with potentially long-term consequences for the infant (Munyaka et al., 2014). These studies suggest that we can begin to distinguish between exposure, transient and long-term colonization.

In addition to dynamics, by sampling broadly, we can further determine the routes of transmission among commensal organisms. Of the transmission routes that pathogens exploit—vertical, airborne, sexual, vector-borne, food-based, water-borne or healthcare-associated transmission—which ones are relevant to commensals? Many studies have surveyed the microbes present in each of these sources, but less research has focused on measuring human exposures and examining the dynamics of colonization. This will be easiest in cases involving discrete exposure events, but transmission may alternatively be fluid, that is to say that microbes are continually circulated within our proximal environments. Understanding these dynamics will assist future public health and environmental efforts to promote the spread of beneficial bacteria, while thwarting those that contribute to dysbioses. Measuring these impacts will undoubtedly benefit from higher resolution, strain-level distinctions, made possible by metagenomic whole microbiome shotgun sequencing.

## Determining transmission routes of human-associated microbiota

**Figure 1:**
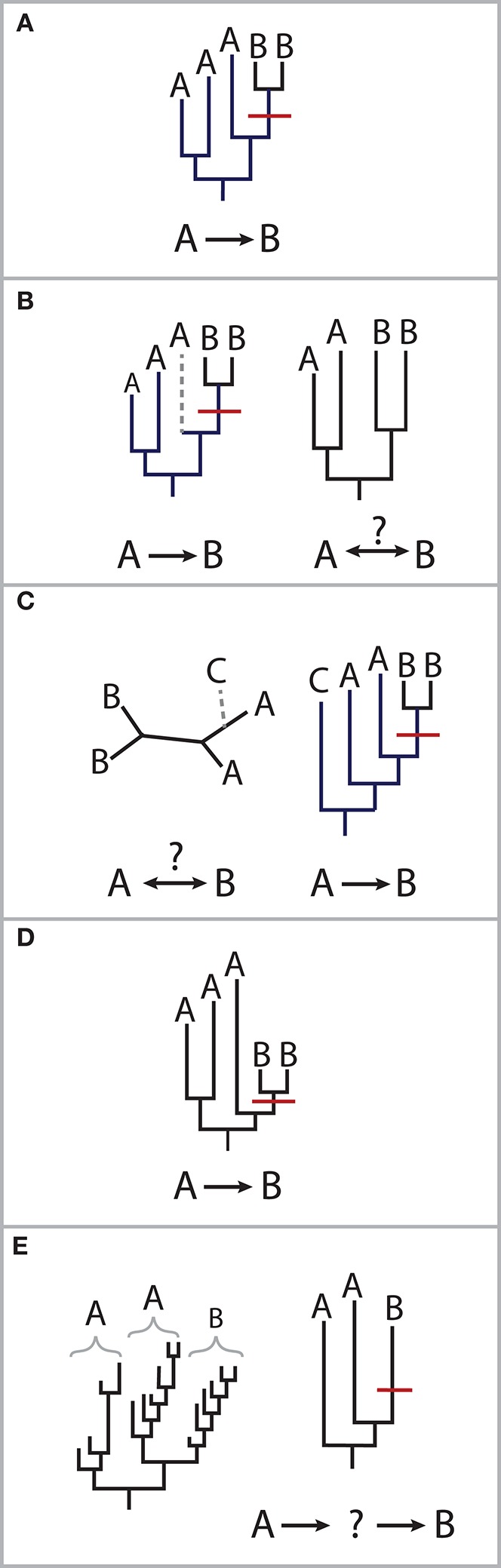
**Scenarios for molecular epidemiology approaches. (A)** Nesting of one individuals' strain lineages within another's supports transmission from the host carrying the ancestral strain to the host carrying the more recently diverged strain, as shown here of a putative transmission event (shown in red) from person A to person B. **(B)** The loss of lineages can affect our ability to determine directionality. Given the same phylogeny in **(A)**, without the gray lineages, it is unclear which person's strains are ancestral. This can occur due to the choice of gene or characterizing fewer strains in an individual than what is present. **(C)** An outgroup helps distinguish transmission direction. Without lineage **(C)**, it is unclear whether **(A)** transmitted strains to **(B)** or vice versa. The inclusion of appropriate control samples can help reduce the likelihood of indirect transmission from an intermediate host or environmental source. In the 1994 case involving HIV, controls were chosen from HIV-infected individuals in the same geography, although not necessarily with the same risk factors (i.e., drug use, sexuality, hemophilia; Metzker et al., 2002). **(D)** Phylogenetic distances may not reflect the timing of transmission. An organism's rate of evolution may depend on factors specific to the individual, such as immunity, diet or genetics, which create different host selective pressures. **(E)** The rate of evolution of the marker gene is important to detect putative direct transmission. Long-term carriage of a microbe with high rates of evolution may result in long branch-lengths, upon which it becomes more difficult to exclude the possibility of indirect transmission.

In 1994, a gastroenterologist was brought to trial for intentionally infecting his girlfriend with HIV-1 virus carried by one of his patients. In order to prove the source of the girlfriend's infection, evidence was sought in the phylogenies of the virus's reverse transcriptase and envelope glycoprotein genes. Virus recovered from her blood was nested within a clade of the patient's, and 28 additional HIV patients from the area were all outgroups to this clade (Metzker et al., 2002). Only the less mutagenic RT sequences were adequate in showing that the strain present in the girlfriend was derived from the patient's HIV infection. This case is a good illustration of the evidence needed to establish transmission: the phylogeny of a gene that captures nested relationships, comprehensive sampling of potential sources to improve the likelihood of observing a direct transmission link, an organism that has an intermediate level of within-host evolution, and a putative transmission mechanism or discrete transmission event. While a transmission link may be impossible to prove conclusively from genomic data alone, these choices impact confidence in determining the timing and directionality of microbial transmission (reviewed in Pybus and Rambaut, 2009; Romero-Severson et al., 2014; Figure 1).

Can molecular epidemiology approaches, typically performed on one species alone, be applied to the diverse communities typical of the human microbiome? Although bacteria mutate less frequently than viral genomes, molecular epidemiology approaches have had some success in inferring the transmission of bacterial pathogens. For example, this was done in the case of the 2001 release of *Bacillus anthracis* in the mail system (Jernigan et al., 2002), as well as in reconstructing the transmission networks of several bacterial outbreaks (reviewed in Gardy et al., 2011; Snitkin et al., 2012; Fricke and Rasko, 2014; Gilchrist et al., 2015). More recently, they have been applied to identify strains of two endogenous human gut bacteria, *Methanobrevibacter smithii* and *Bacteroides thetaiotaomicron* shared between sets of twins (Faith et al., 2013).

Finding signals of transmission within metagenomic data may be made easier if there is more evolutionary divergence between samples. In the absence of high mutation rates, long-term carriage can result in greater within-host evolution, making it easier to reconstruct phylogenies. *Helicobacter pylori, Mycobacterium tuberculosis* and *Burkholderia dolosa*, a long-term infection associated with cystic fibrosis, are several bacteria that have accumulated an adequate number of mutations to track transmission across individuals (Falush et al., 2001; Gardy et al., 2011; Lieberman et al., 2011). Evidence that many commensal microbes have long-term residence in the gut and skin, (Faith et al., 2013; Schloissnig et al., 2013; David et al., 2014b; Oh et al., 2014), possibly dating back to birth (Dominguez-Bello et al., 2010), lends credence to applying molecular epidemiology approaches to a range of bacterial species in the human microbiome.

To attain the genomic resolution necessary to infer transmission, these studies have all relied on whole genome sequencing of cultured isolates. Applying this method to the greater variety of bacteria in the human microbiome would have limited scalability and would be restricted to culturable organisms. Single-cell techniques offer a way to circumvent culture limitations and the problems associated with genotyping strains that arise from short-read sequencing (discussed below). These can be technically challenging and costly, as hundreds of single-cell genomes per individual sample would be required to capture the diversity of strains of multiple species that are routine sampled using untargeted metagenomic sequencing. Rather, with short-read metagenomic sequencing, genomes of many species may be acquired from a single sample, providing the raw data to infer transmission networks.

Comprehensive, metagenomic data is inherently more complex because it involves sequencing all bacterial, viral, and eukaryotic (including human) DNA present in a sample simultaneously, and the linkage of reads to each particular genome is lost during this process. To make sense of a diverse set of metagenomic reads, sequences must be aligned to reference genomes or *de novo* assembled draft genomes. Previous efforts to identify organisms this way have had mixed results: only 67% of culture-positive samples for Shiga-toxinogenic *E. coli* O104:H4 were identified by alignments to a *de novo* assembled genome of this organism (Loman et al., 2013). Disentangling genotypes down to the strain-level may be more complicated than this example for several reasons: genotyping strains from many species requires adequate coverage of each species, which may be hard to attain with the highly uneven distribution of species in a sample; individuals typically carry a handful of closely related strains within a species (Faith et al., 2013; Schloissnig et al., 2013; Oh et al., 2014); recombination may occur between closely related strains (Falush et al., 2001); and transmitted organisms are likely to resemble organisms already present in the gut (David et al., 2014b; Krebes et al., 2014). Yet, in order to get closer to proving transmission, we need an organismal resolution more fine-grained than species. The challenge will be to unambiguously genotype strains present within each individual.

## Achieving strain-level accuracy

Metagenomic data is more appropriate for strain-calling than 16S rRNA amplicon data. The main reason is that metagenomic sequencing requires relatively few rounds of DNA amplification, compared to 16S amplicon sequencing, thus reducing the chance that PCR and sequencing errors are mistaken as genuine single nucleotide polymorphisms (SNPs). Although there are various computational methods available to address this issue with 16S amplicons, they usually carry the unintended consequence of a loss of resolution (Edgar et al., 2011; Quince et al., 2011; Schloss et al., 2011; Bokulich et al., 2013; Preheim et al., 2013). There is a cost to attaining higher resolution data. The main challenge in defining strains from short-read sequencing is that SNP frequencies in the genome that can be used to distinguish between recently diverged strains do not appear more than once per 100–250 bp, which is the typical read length of ubiquitous high-throughput short-read sequencers. Therefore, metagenomic sequencing requires far more reads per sample to attain adequate coverage and depth of a genome required for phasing and distinguishing between strains. Also, rather than using standard analytical pipelines that exist for 16S, such as QIIME (Caporaso et al., 2010), there are no universally accepted methods for strain-level characterization from metagenomic data.

There have been several proposed strain-calling methods (Table 1), though most of these methods stop short of actually genotyping strains and instead focus on shared genomic features across samples, with the exception of ConStrains method which results in strain genotypes and their abundances (Luo et al., 2015). These methods generally rely on aligning reads to reference genomes, although this may be insufficient for unique samples for which reference genomes do not yet exist. Several methods overcome this limitation, enabling *de novo* assembly of genomes across metagenomic samples (Boisvert et al., 2012; Pell et al., 2012; Howe et al., 2014; Cleary et al., 2015). The Latent Strain Analysis method (Cleary et al., 2015) is notable because species of very low abundance (as low as 0.00001% in one case) distributed across many samples can be successfully assembled.

**Table 1:** **Methods for strain characterization from metagenomic data**.

**DNA regions**	**Considerations**
SNPs within core or species-specific genes (Schloissnig et al., 2013; Oh et al., 2014; Ahn et al., 2015; Luo et al., 2015)	Methods either resolve genotypes or examine co-occurrences of SNPs. Genes may have different rates of evolution. Alignments may be difficult in the presence of closely related species.
Non-overlapping 1 kb windows (Franzosa et al., 2015)	Windows may contain a mix of horizontally transferred and core genomes. Limited phylogenetic analysis.
Copy-number variations of genes (Greenblum et al., 2015)	Rates of mutation may be harder to estimate.
Junctions of horizontally transferred regions and core genome (Raveh-Sadka et al., 2015)	Co-occurrence of transferred regions may change rapidly. Assembly may be difficult at repetitive regions common at HGT junctions. HGT may obscure phylogenetic patterns useful for inferring transmission.
CRISPR spacer comparisons (Raveh-Sadka et al., 2015)	Rates of spacer acquisition may be harder to estimate. Identifying source of mobile element may be difficult.

Both assembly- and alignment-based methods for genotyping strains require high depth and even coverage of each genome or DNA segment being analyzed. This is easily attainable for bacteria-rich samples such as the gut, where the predominance of bacteria results in relatively little human DNA. Conversely, in bacteria-poor environments that may be important for the study of transmission, such as the skin, a large fraction of the DNA sequenced, upwards of 90%, is from human cells (Human Microbiome Project Consortium, 2012). A greater amount of sequencing is therefore required to achieve adequate coverage of bacterial genomes. Additionally, the right-skewed abundance distributions of bacteria in some human body sites, such as the gut, contributes to this problem, such that large increases in sequencing depths are required to adequately cover lowly abundant organisms (Ni et al., 2013; Wendl et al., 2013). Since the costs associated with increased sequencing may soon cease to be a limiting factor and out-of-bag computational methods will become available, strain-level analysis may become as commonplace as marker gene analysis is today.

Newer sequencing approaches that produce longer read lengths may alleviate the need for such high sequencing depth and may allow for strain comparisons that utilize larger genomic regions than outlined in Table 1 or even full genomes. The minION, made by Oxford Nanopore Technologies, has provided strain-level data in outbreak settings, specifically of Ebola (Quick et al., 2015) and *Salmonella* enterica serovar Enteritidis (Quick et al., 2016) that was used for transmission mapping. It has yet to be used to simultaneously examine the transmission of the numerous members of complex bacterial communities. Other experimental alternatives achieve synthetic long read lengths by manipulating amplification protocols to provide additional linkage information. For example, single kb-length molecules can each be sorted into a well, sheared, identically barcoded, and later assembled into one high fidelity scaffold (Kuleshov et al., 2014). Although this approach is lower throughput, it has been has been used together with short-read sequencing to improve scaffolding of highly-fragmented assemblies that can arise from *de novo* sequencing (Sharon et al., 2015). Proximity ligation is another experimental manipulation that uses Hi-C sequencing, i.e., intra-genome crosslinking, to link read-pairs arising from a single DNA molecule and has also been successfully used to genotypes strains within complex microbiome samples (Beitel et al., 2014; Burton et al., 2014). Although these technologies have been used on a very limited number of samples, they hold tremendous promise for achieving high confidence genotypes required to deconvolve chains of microbial transmission in complex communities.

## Frontiers of microbial transmission studies in health and the environment

We are now in an age where it is possible to engineer the microbiome to achieve therapeutic outcomes and modify our environments. Live bacterial therapeutics are already being used to treat *Clostridium difficile* infections (Kassam et al., 2013; Olle, 2013), and bioengineered therapeutics are on the horizon. Synthetic strains could be modified for a variety of applications within the human body, for enzyme replacement, disease prevention, and diagnostic capabilities; or in the environment, for hazardous material remediation, pest control, and drought prevention. High confidence strain-tracking will be essential to gauge the dispersal of artificially introduced organisms. A handful of studies are beginning to track microbial strains, for example, after intentional inoculation. These include monitoring the infant gut microbiome throughout its development (Sharon et al., 2013); examining the donor and recipients of fecal microbiome transplants; and examining transmission in close-knit agrarian communities as part of the Fiji Community Microbiome Project (www.FijiCOMP.org).

**Figure 2:**
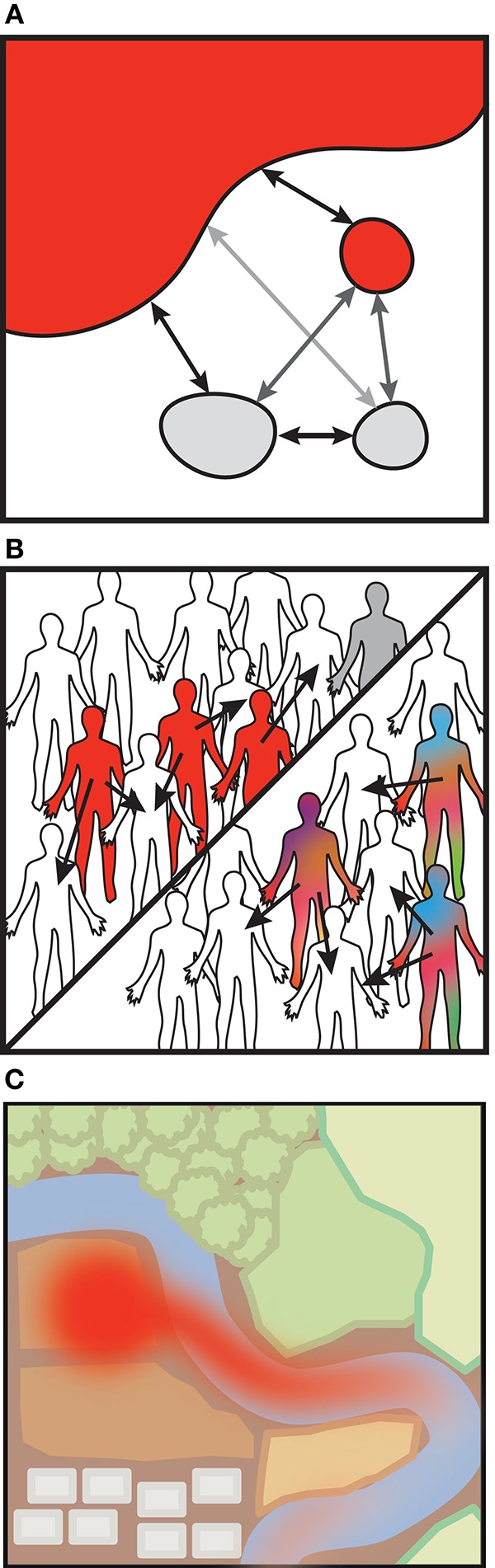
**Modeling bacterial transmission. (A)** Metapopulation models. Change in island occupancy, by a microbe perhaps, is modeled as a function of migration (*m*) and an extinction rate (*e*). Other considerations such as a distance-based probability of infection may modify *m.*
$\frac{dP}{dt}=mP\;\left(1-P\right)-eP$ **(B)** Susceptible-Infected-Resistant (SIR) models (with or without strain dynamics). Susceptible (S) individuals may become infected (I) and can recover and become immune. SIR models are similar to metapopulation models in that infection rate (β) is akin to migration between islands, as recovery (γ) is akin to extinction in the metapopulation model. Variations may include demographic processes, infection processes (latency, carriage), and alternative hosts or vectors.
$$\frac{dS}{dt}=-\beta SI$$
$$\frac{dI}{dt}=\beta SI-\gamma I$$
$\frac{dR}{dt}=\gamma I$ SIR models that incorporate within-host evolution of specific strains typically are nested models that account for individuals' infection composition. **(C)** Landscape fate-and-transport (F&T) models. F&T models estimate microbial abundances rather than a dichotomous infection status. The models stem from traditional advection-dispersion equations. Landscape features such as the surface porosity or water flow can be incorporated.
$$\frac{\partial C}{\partial x}=D\frac{\partial^{2}C}{\partial^{2}x}-\nu \frac{\partial C}{\partial x}$$

Beyond characterizing strains within isolated samples, longitudinal strain-level data would allow us to approach the question posed earlier in this review: how does transmission impact daily or punctuated shifts in our microbiomes? While it may be straightforward to measure the impacts of transmission after a discrete event, in cases where transmission is continuous between source and sink, estimating rates of dispersal and transfer will be nontrivial. Mathematical models originally intended to capture animal movements or pathogen transmission may be adapted to account for the strain dynamics within diverse microbial communities. Metapopulation models, for example, describe environmental niches as “islands” between which organisms can migrate (Levins, 1969; Hanski, 1998). In the simplest of such models, unoccupied islands become occupied by the influx of bacteria from occupied islands, and extinction events in occupied islands may leave them unoccupied (Figure 2A). In the case of the human microbiome, these “islands” could be different individuals or body sites (Costello et al., 2012). Ecological disease models are similar to metapopulation models, though rather than colonizing islands, individuals are infected (Figure 2B). They differ in that individuals may transition from susceptible (S) to infected (I) classes, but may also transition to recovered classes (R) where they are no longer susceptible (Anderson and May, 1979). These SIR models come in a wide range of flavors and can be deterministic, stochastic, agent-based or spatially explicit, but they generally monitor the status of infected or uninfected units. Although infection will differ than colonization, these models provide analytical frameworks to start testing transmission rates and mechanisms.

Alternatively, there are models which account for the abundances of organisms within individuals or across a landscape, rather than their mere presence. Within-host pathogen models build upon the SIR model framework and track the abundances of a small number of strains resulting from mutation and local selection, as from immune pressure (Grenfell et al., 2004; Mideo et al., 2008; Figure 2B). Within-host and population-based SIR models can be nested as these dynamics may interact at different levels (reviewed in Mideo et al., 2008). Environmental fate-and-transport models similarly model pathogen abundances across landscape features and can incorporate environmental conditions that impact dispersal (reviewed in Brookes et al., 2004; Benham et al., 2006; Figure 2C). Fate-and-transport models may also be linked to SIR models to quantify bacterial exposures (Eisenberg et al., 2002). There is ample opportunity to apply these techniques toward understanding microbiome-related transmission.

How can microbiome data be incorporated into transmission models? First, models designed for one microbial organism must be adapted to account for many. Parameterizing such models may be challenging given the broad differences in transmission observed between even closely related strains (Lee et al., 2013). Second, models of microbial communities may need to account for microbial interactions. Models of multiple pathogens show that complex dynamics can result from pathogen interactions (Rohani et al., 2003), and there are examples to suggest that this will be true for commensal organisms as well (David et al., 2014b; Hsiao et al., 2014; Seedorf et al., 2014). Lastly, we will also need to transform such models to accommodate compositional data. SIR models of more than one pathogen typically assume that measurements of each pathogen are independent (Rohani et al., 2003). Whereas counting microbes is technically challenging, microbial community measurements often reflect relative abundances of bacteria rather than absolute abundances. Although there are some methods that can escape this limitation (Friedman and Alm, 2012; Kurtz et al., 2015), we still lack principled methods to normalize time series compositional data. Figuring out how to incorporate multiple species into models of microbial transmission will be challenging but is a next logical step in our understanding of these communities.

In the near future, we predict that strain-tracking will become increasingly important, whether for epidemiology, forensics, environmental monitoring, or diagnostics. Metagenomics is currently the most straightforward and affordable data that can be used to track strains, and will likely be the primary source of those data in the near term. Despite the widespread availability of metagenomic sequencing, off-the-shelf methods to identify and evaluate the distribution of strains are still needed. In time, refinements will be made to determine what study design, sample preparation and sequencing depth are needed to substantiate claims of specific transmission chains. When that time comes, we may be able to quantify the role of commensal transmission in Crohn's disease, autoimmune disease, obesity and other microbiome-linked pathologies.

## Author contributions

All authors listed, have made substantial, direct and intellectual contribution to the work, and approved it for publication.

## Funding

We would like to thank the Neil and Anna Rasmussen Foundation for their support.

### Conflict of interest statement

The authors declare that the research was conducted in the absence of any commercial or financial relationships that could be construed as a potential conflict of interest.
